# Melatonin improved the outcomes of women with ART: a systematic review and meta-analysis of randomized trials

**DOI:** 10.3389/frph.2025.1680984

**Published:** 2025-09-23

**Authors:** Yilin Wu, Wenjie Huang, Li Tang, Yuelin Feng, Hongqing Chen, Mingxin Pan, Jingrong Peng, Chen Li, Huawei Wang

**Affiliations:** ^1^ Reproductive Genetics Department, The First Affiliated Hospital of Kunming Medical University, Kunming, Yunnan, China; ^2^ Department of Reproductive Medicine, Guangzhou Women and Children’s Medical Center Liuzhou Hospital, Liuzhou, Guangxi, China; ^3^ Department of Gynecology, The First Affiliated Hospital of Kunming Medical University, Kunming, Yunnan, China

**Keywords:** melatonin, clinical pregnancy rate, assisted reproductive technologies (ART), RCT, embryo quality, systematic review and meta-analysis

## Abstract

**Objective:**

To systematically evaluate whether the melatonin supplementation could improve the embryo development and pregnancy outcomes of infertile women undergoing assisted reproductive technologies (ART).

**Methods:**

This systematic review and meta-analysis followed the PRISMA guidelines and was prospectively registered in PROSPERO (CRD420251003042). The randomized controlled trials (RCTs) published before March 5, 2025 are included to evaluate the efficacy of melatonin on infertile women undergoing ART. Eligible studies reported at least one embryo development or pregnancy-related outcome. Primary outcome was clinical pregnancy rate; secondary outcomes including oocyte yield, fertilization rate, MII oocyte number, and high-quality embryo formation. Subgroup analyses were conducted based on stimulation protocols, melatonin dosage, and population characteristics. Risk of bias was assessed using the Cochrane Risk of Bias tool, and pooled effect sizes were calculated using fixed- or random-effects models depending on heterogeneity. Totally, eleven RCTs with a total of 1,481 participants were analyzed here.

**Data sources:**

PubMed/MEDLINE, Embase, and Cochrane Library.

**Results:**

Melatonin supplementation significantly improved clinical pregnancy rate (OR = 1.59, 95% CI: 1.22–2.07). Regarding embryo development, melatonin significantly increased the number of high-quality embryos (MD = 0.43, 95% CI: 0.07–0.79), MII oocyte (SMD=0.99, 95% CI: 0.29–1.69), and fertilization rates (OR = 1.32, 95% CI: 1.01–1.73). No significant difference was observed in oocyte yield (SMD = 0.45, 95% CI: −0.04 to 0.94). Subgroup analysis revealed enhanced clinical pregnancy outcomes with ≤3 mg/day melatonin and under GnRH-a long protocols. Moderate to high heterogeneity was observed in some secondary outcomes, with publication bias suggested for the MII oocyte outcome.

**Conclusions:**

Melatonin supplementation may improve intermediate outcomes such as fertilization, embryo quality, and clinical pregnancy rates in women undergoing ART. With a favorable safety profile, it could be a low-cost adjunct for selected patients, though standardized guidelines are lacking and large-scale RCTs are needed to clarify long-term effects.

**Systematic Review Registration:**

https://www.crd.york.ac.uk/prospero/display_record.php?ID=CRD420251003042, PROSPERO CRD420251003042.

## Introduction

Infertility has become an increasingly prevalent global public health concern in recent years. According to the data of World Health Organization (WHO), approximately 10% to 15% of couples with reproductive age worldwide are affected by infertility (1). Beyond its physiological implications for women, infertility also imposes significant psychological, social, and familial burdens (2, 3). With ongoing advancements in assisted reproductive technologies (ART), *in vitro* fertilization (IVF) has become the cornerstone of infertility treatment. Current strategies primarily aim to improve embryo quality and optimize endometrial receptivity (4, 5). However due to the delayed childbearing, high levels of psychological stress, mental health issues including anxiety, insomnia, and depression, which could cause the the poor oocyte quality with the higher level of reactive oxygen species (ROS) and might cause a plausible poor oocyte quality, lower fertilization and pregnancy rates, and failed ART (6). Further enhancement of embryo viability and pregnancy outcomes remains a major challenge in reproductive medicine. As such, the development of safe and effective adjunctive interventions has emerged as a critical research priority.

Melatonin, an indoleamine hormone secreted by the pineal gland, is well known for regulating circadian rhythms and exerting potent antioxidant effects (7, 8). As an antioxidant, melatonin has been proposed as a promising therapeutic agent for improving oocyte quality and reproductive outcomes. It is thought to mitigate oxidative stress in the ovaries, thereby enhancing the quality of oocytes (9). In ovarian granulosa cells, melatonin has also been shown to inhibit autophagy via modulating of signaling pathways such as PI3K–Akt–mTOR, offer further cytoprotective effects (10).

Although several randomized controlled trials (RCTs) have demonstrated that melatonin supplementation might increase the proportion of metaphase II (MII) oocytes of ART (11), enhance endometrial thickness and clinical pregnancy rates (12), and reduce oxidative stress in follicular fluid (13), however, other study failed to observe significant improvements in clinical pregnancy or live birth rates (11). These inconsistencies may be due to melatonin dosage, treatment duration, or the concomitant use of adjunctive agents such as myo-inositol, thus which limited cross-study comparability and generalizability (14–16). Despite some encouraging findings, current evidences on the efficacy of melatonin in ART are remain inconsistent. Yet, several meta-analyses also have yielded conflicting results, while one analysis suggested improvements in clinical pregnancy rate and oocyte quality without significant benefit for live birth rates (17), another work highlighted considerable uncertainty across key outcomes (18), Mejlhede et al. (6) observed the increased mature oocyte counts with melatonin, however, the clinical pregnancy rates did not significantly improve.

These discrepancies underscore the necessary for an updated and methodologically rigorous meta-analysis. Previous reviews have been restricted by the limited available articles, incomplete outcome reporting, and insufficient heterogeneity assessment. Therefore, the present study aims to provide a comprehensive synthesis of the evidence through a systematic review and meta-analysis, evaluating the impact of melatonin on embryo development and pregnancy outcomes in women undergoing ART. Additionally, this study seeks to investigate potential effect modifiers, such as melatonin dosage, patient characteristics, and COS protocols, thereby offering more clinically actionable insights.

## Materials and methods

This review was conducted and reported in accordance with the Preferred Reporting Items for Systematic Reviews and Meta-Analyses (PRISMA) guidelines. The protocol was prospectively registered in the PROSPERO database (registration number: CRD420251003042). We included all randomized controlled trials (RCTs) that evaluated the effects of melatonin on oocyte quality and pregnancy outcomes in women with infertility, and reported at least one embryo- or pregnancy-related outcome. Eligible participants were women of any age undergoing assisted reproductive treatment and receiving melatonin supplementation. The primary outcome was clinical pregnancy rate. Secondary outcomes included the number of oocytes retrieved, fertilization rate or fertilized oocytes, and the rate or number of high-quality embryos or blastocysts. Studies were excluded if they were quasi-randomized trials, cohort studies, case-control studies, case reports, animal or *in vitro* experiments, or if they lacked sufficient statistical data for the outcomes of interest. In some instances, investigators discussed outcomes in their conclusions without providing extractable data; in such cases, we cited the original interpretation but did not include it in the pooled analysis. A comprehensive search was performed in PubMed/MEDLINE, Cochrane Library, and Embase for studies published in English up to March 5, 2025. The search strategy included combinations of the following keywords: “melatonin”, “subfertility”, “fertilization *in vitro*”, “premature ovarian failure”, and “ovarian reserve”. The complete search terms are provided in Supplementary Table S1.

### Study selection and data collection process

Two reviewers (W.Y. and H.W.) independently screened the titles and abstracts of all retrieved articles. Studies deemed irrelevant by both reviewers were excluded. The screening process were blinded to author names, institutional affiliations, journal titles, and study outcomes. Any disagreements were resolved through discussion with a third reviewer. Subsequently, four reviewers (W.Y., H.W., C.H., and W.H.) independently assessed the full texts of potentially eligible articles according to the predefined inclusion criteria and evaluated their methodological validity. All extracted data were cross-checked for accuracy, and discrepancies were resolved by consensus.

The extracted data included: first author, year of publication, sample size, number of participants in each group, stimulation protocols, intervention details, control measures, and reported outcomes. These characteristics were summarized in a comparative table (Table 1) for ease of cross-study comparison.

**Table 1:** Characteristics of the included studies on melatonin supplementation in assisted reproduction.

Author, year, country	Sample size	COS protocol	protocol	Control arm	Period spanning	Outcomes
Sadeghpour et al. Iran (19)	*n* = 68, *t* = 34, con = 34	GnRH-a long	Melatonin, 5 mg/d, taken orally during IVF protocol.	placebo	Starting on the 3–5th day of the previous menstrual cycle	Number of retrieved oocytes Number of MII oocytes Fertilization Rate High quality embryo count
Pilehvari et al. lran (20)	*n* = 320, *t* = 160, con = 160	GnRH-a long and GnRH-a short	combination of melatonin and metformin as an insulin sensitizer (3 mg and 500 mg, respectively) three times a day.	Metformin, 500 mg	14 days (from the luteal phase of the cycle before the stare of gonadotropin)	Number of retrieved oocytes Clinical pregnancies rate Metaphase II rate High quality embryo rate
Fernando et al. Australia (21)	*n* = 150, *t*(2 mg) = 41, *t*(4 mg) = 39, *t*(8 mg) = 40, con = 40	GnRH-A	2 mg melatonin capsule twice per day (4 mg/day total) 4 mg melatonin capsule twice per day (8 mg/day total) 8 mg melatonin capsule twice per day (16 mg/day total)	Placebo capsule twice per day;	NR	Number of retrieved oocytes Number of fertilized oocytes High quality embryo count
Jahromi et al. Iran (15)	*n* = 80, *t* = 40, con = 40	GnRH-a long	Melatonin, 3 mg/night, taken orally from the 5th day of the menstrual cycle prior to the cycle that was planned for ovarian stimulation.	Placebo	50 days	Number of MII oocytes, High-quality embryo rate Clinical pregnancies rate
Pacchiarotti et al. Italy (11)	*n* = 331, *t* = 165, con = 166	GnRH-a long	Melatonin(3 mg), MI (4,000 mg), folic acid (400 mcg)	MI (4,000 mg), folic acid (400 mcg)	14 days(from the first day of the cycle until 14 days after embryo transfer)	Number of retrieved oocytes Metaphase II rate Clinical pregnancy rate high-quality embryo rate
Batıoğlu et al. Turkey (22)	*n* = 85, *t* = 40, con = 45	GnRH-a long	Melatonin, 3 mg/d	No treatment		Number of retrieved oocytes Number of MII oocytes Fertilized oocyte count High-quality embryo count Clinical pregnancy rates
Rizzo et al. Italy (23)	*n* = 65, t = 32, con = 33	GnRH-a long	2 g myo-inositol twice a day, 200 mg folic acid, 3 mg melatonin	2 g myo-inositol twice a day, 200 mg folic acid	Administrated continuously from the day of GnRH administration	Number of retrieved oocytes Number of MII oocytes Fertilized oocyte rate Clinical pregnancy rates high-quality embryos
Espino et al. Spain (13)	*n* = 30, t(3 mg) = 10, t(6 mg) = 10, con = 10	GnRH-A	3 mg melatonin was taken one hour before sleep 6 mg melatonin was taken one hour before sleep	No treatment	Starting on the day of GnRH administration	Number of retrieved oocytes Number of MII oocytes rate Fertilized oocyte rate Clinical pregnancies rate
Eryilmaz et al. Turkey (24)	*n* = 60, *t* = 30, con = 30	GnRH-a long	3 mg melatonin was teken at 10:00–11:00pm	No treatment	3 days	Number of retrieved oocytes Fertilized oocyte rate Number of MII oocytes High-quality embryo rate Clinical pregnancies rate
Akter et al. Bangladesh (14)	*n* = 74, *t* = 40, con = 34	GnRH-a short	3 mg melatonin was taken before sleep,combination with 5 mg letrozole from day2 of menstrual cycle.	5 mg letrozole from day 2 of menstrual cycle.	8 weeks	Number of MII oocytes Clinical pregnancies rate
Mokhtari et al. Iran (12)	*n* = 198, *t*-98, con = 100	IUI	3 mg melatonin combination with clomiphen citrate 50 mg	Placebo combination with clomiphen citrate 50 mg	NR	Clinical pregnancies rate

Data include the first author, year of publication, country, sample size and population type, controlled ovarian stimulation (COS) protocol, intervention and control arms, timing of intervention, and reported reproductive outcomes (e.g., number of retrieved oocytes, number of MII oocytes, fertilization rate, high-quality embryo rate, clinical pregnancy rate). COS, controlled ovarian stimulation; MII, metaphase II; PCOS, polycystic ovary syndrome; DOR, diminished ovarian reserve.

### Risk of bias and quality assessment

The risk of bias was independently assessed by four reviewers (W.Y., H.W., C.H., and W.H.) using the Cochrane Handbook methodology and the Cochrane Risk of Bias Tool. The assessment covered the following domains: random sequence generation, allocation concealment, blinding of participants and personnel, blinding of outcome assessors, incomplete outcome data, selective reporting, and other potential sources of bias. Each domain was rated as having a “low”, “high”, or “unclear” risk of bias. Based on the overall assessment across these domains, studies were categorized as having low, unclear, or high overall risk of bias.

### Statistical analysis

Statistical analyses were performed using the meta package in R (version 4.4.2). Heterogeneity was assessed using Cochran's *Q* test and the *I*² statistic. An *I*^2^ > 50% or a *p*-value < 0.05 was considered indicative of substantial heterogeneity. When heterogeneity was present, subgroup analyses and meta-regression were conducted to explore potential sources, such as variation in stimulation protocols or participant characteristics. Funnel plots were used to qualitatively evaluate publication bias. Sensitivity analyses were performed to assess the robustness of the pooled results. Both random-effects and fixed-effects models were applied as appropriate, with the random-effects model used for the primary analysis in the presence of heterogeneity.

Subgroup analyses were conducted based on stimulation protocols, melatonin dosage, and participant characteristics, including diminished ovarian reserve (DOR), polycystic ovary syndrome (PCOS), and infertility type. For each subgroup, we reported effect estimates (odds ratios [OR], mean differences [MD], or standardized mean differences [SMD]), along with 95% confidence intervals and I² statistics. Sensitivity analyses were conducted by excluding studies with a high risk of bias or those that had a disproportionate influence on the overall estimates.

## Results

### Study selection

**Figure 1:**
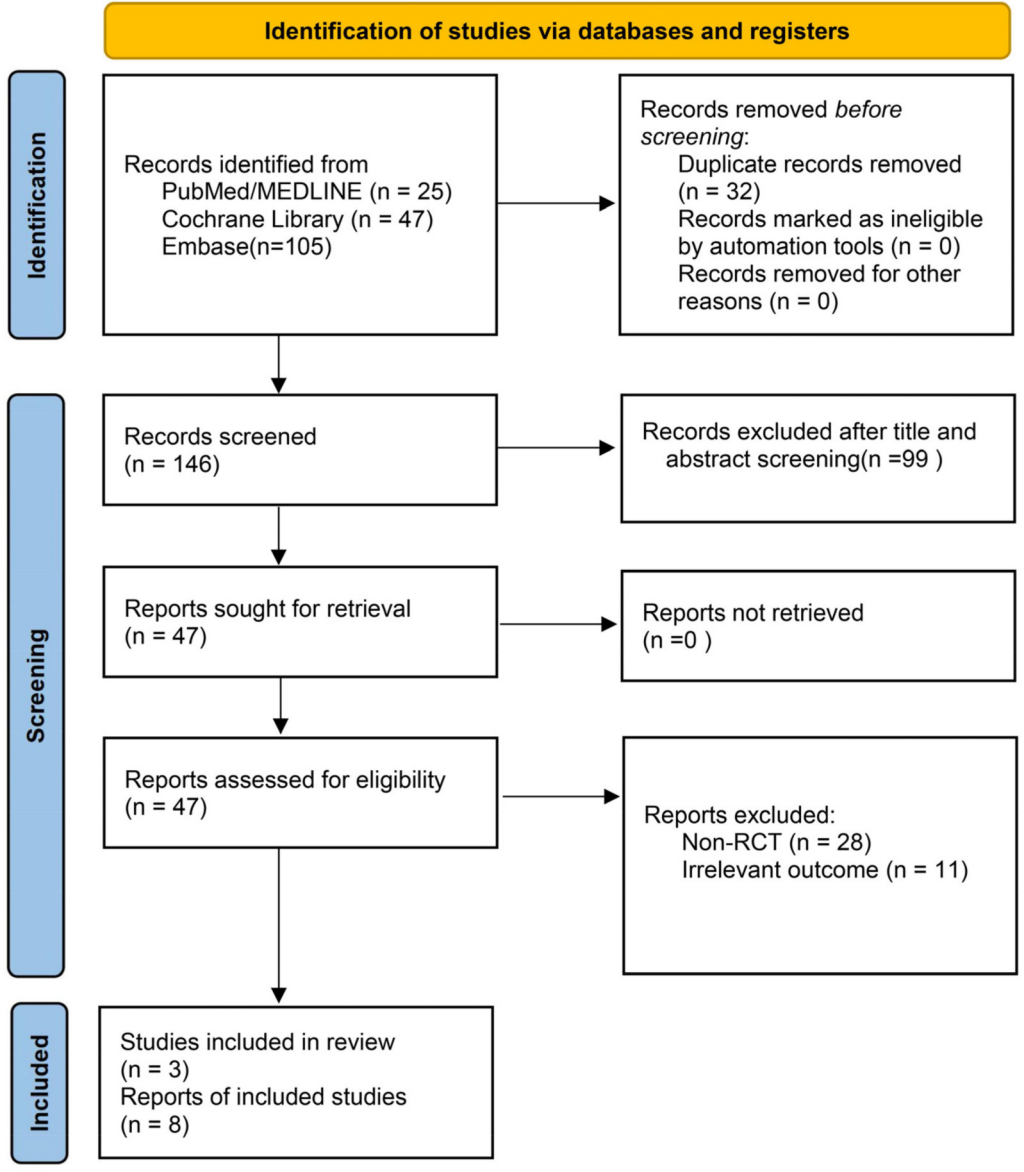
PRISMA flow chart for a systematic review and meta-analysis of clinical pregnancy rate following melatonin treatment in women undergoing assisted reproduction.

A total of 178 records were retrieved from PubMed with the process described in Figure 1, Embase, and the Cochrane Library. After automatic deduplication using Endnote X9, 32 additional duplicate records were manually removed. Title and abstract screening excluded 99 clearly irrelevant studies. Full texts of 47 potentially eligible articles were assessed. Among them, 3 literatures belonged to meta-analyses works, 1 literature was assigned to narrative review, 28 papers were excluded due to non-RCT designs, and 11 works were removed for lacking relevant outcome data. Ultimately, 8 studies met the predefined inclusion criteria were analyzed here. Additionally, 3 eligible studies were identified through citation tracking of existing reviews and meta-analyses, resulting in a final total of 11 studies included in the quantitative synthesis (11–15, 19–24).

The risk of bias assessment is presented in Supplementary Figure S1 and Table 2. All included studies were evaluated using the Cochrane Risk of Bias Tool. Most studies demonstrated low risk in random sequence generation and allocation concealment. However, three studies exhibited potential bias in the blinding of participants, personnel, and outcome assessment. As a result, the overall risk of bias across studies was judged to be moderate. Notably, three studies were categorized as high risk, primarily due to inadequate blinding procedures and insufficient outcome assessment measures. Table 1 summarizes the key characteristics of the included studies, including sample size, controlled ovarian stimulation (COS) protocols, study design, control group details, and treatment duration.

**Table 2:** Risk of bias assessment for included RCTs using the RoB 2 tool (domain-level).

Study	D1	D2	D3	D4	D5	Overall
Sadeghpour et al. (19)	Low	Low	Low	Low	Low	Low
Pilehvari et al. (20)	Low	Low	Low	Unclear	Low	Unclear
Fernando et al. (21)	Low	Low	Low	Low	Unclear	Unclear
Jahromi et al. (15)	Low	Low	Low	Low	Low	Low
Pacchiarotti et al (11)	Low	Low	Low	Unclear	Low	Unclear
Batioglu et al. (22)	Unclear	High	Low	Low	Low	High
Rizzo et al. (23)	Low	Unclear	Low	Unclear	Low	Unclear
Mokhtari et al. (12)	Low	Low	Low	Low	Low	Low
Espino et al. (13)	Low	High	Low	Unclear	Low	High
Eryilmaz et al. (24)	Low	Unclear	Low	Unclear	Low	Unclear
Akter et al. (14)	Low	High	High	Unclear	Low	High

D1: Bias arising from the randomization process.

D2: Bias due to deviations from intended interventions.

D3: Bias due to missing outcome data.

D4: Bias in measurement of the outcome.

D5: Bias in selection of the reported result.

### Clinical pregnancy rate

**Figure 2:**
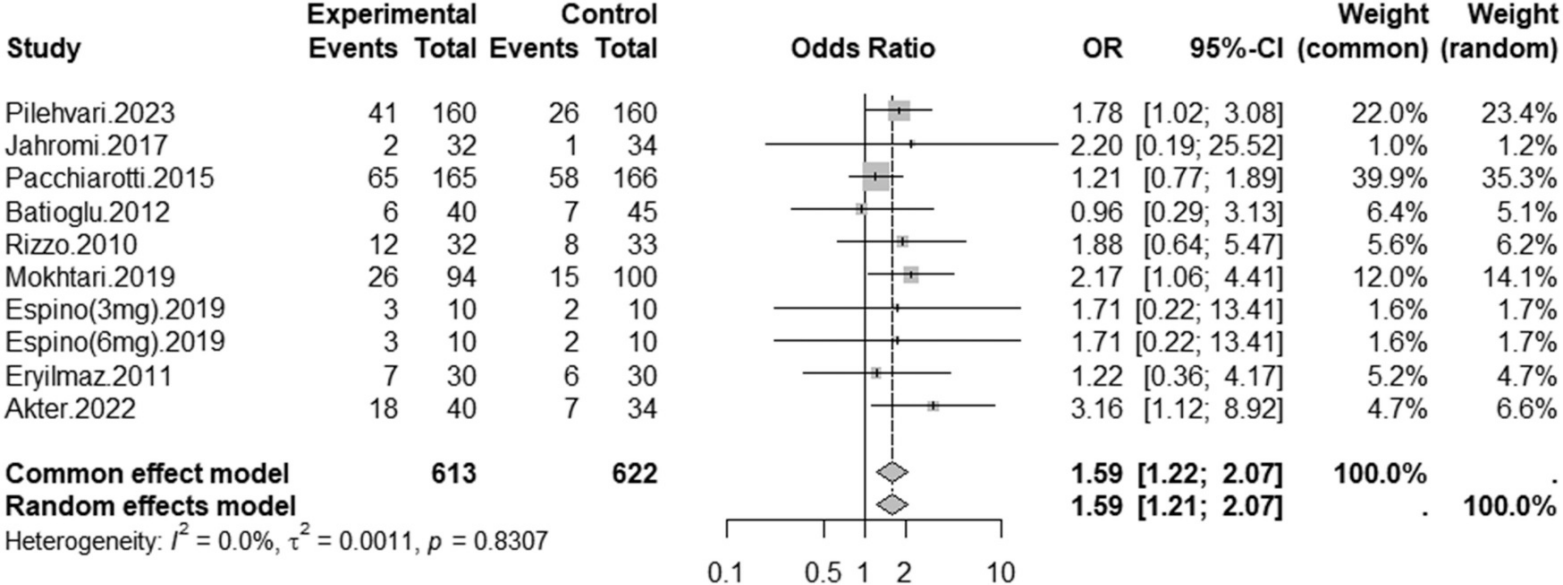
Forest plot of odds ratios (OR) for clinical pregnancy rate following melatonin treatment in assisted reproduction. The plot summarizes the results of individual studies and the overall effect estimate using both common and random effects models.

Figure 2 presents the meta-analysis of clinical pregnancy rate. Nine studies involving a total of 1,235 participants (613 in the melatonin group and 622 in the control group) were included. The pooled analysis showed that melatonin supplementation significantly improved clinical pregnancy rates by comparing that with control group (OR = 1.59, 95% CI: 1.22–2.07), reflecting a statistically significant 59% increase in the odds of clinical pregnancy. Heterogeneity was negligible (*I*^2^ = 0.0%, *p* = 0.8307), supporting the use of a fixed-effects model. Visual inspection of the funnel plot did not suggest substantial publication bias (Supplementary Figure S3E).

### Number of retrieved oocytes

**Figure 3:**
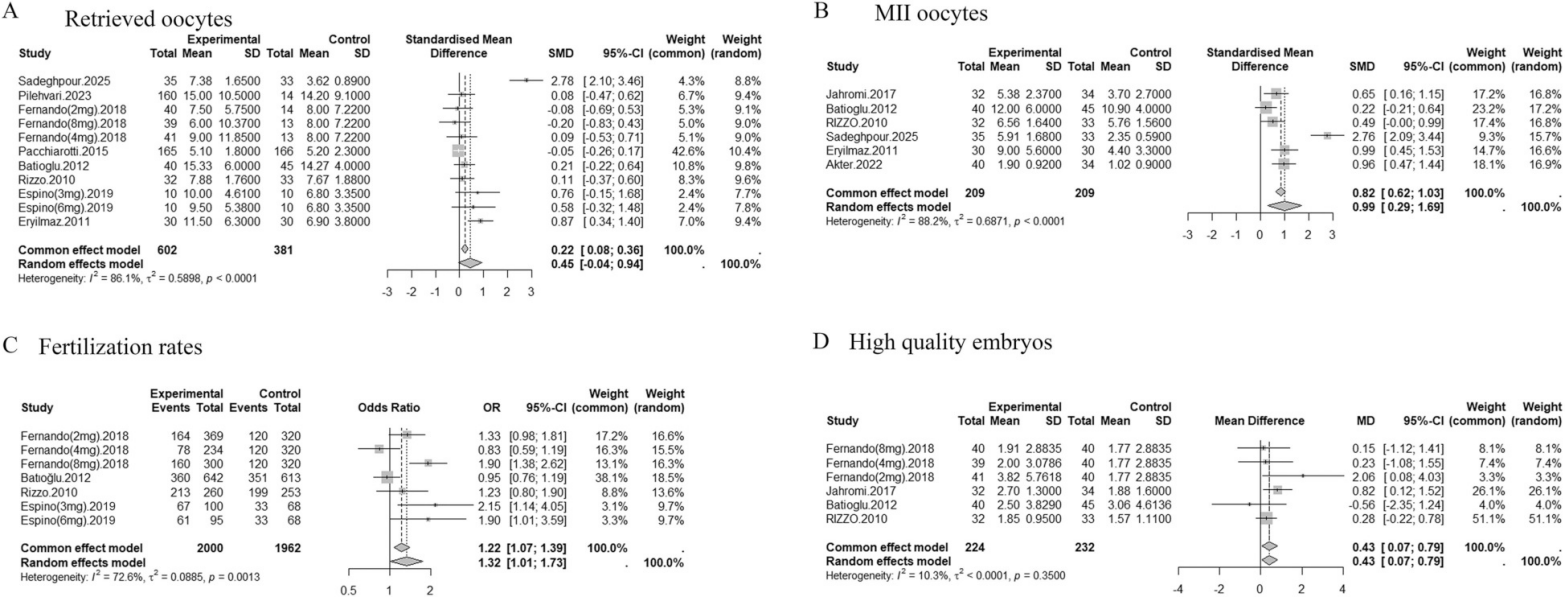
Forest plots of the effects of melatonin treatment on oocyte and embryo outcomes in assisted reproduction. **(A)** Number of retrieved oocytes (SMD). **(B)** Number of MII (metaphase II) oocytes (SMD). **(C)** Number of fertilized oocytes (OR). **(D)** Number of high-quality embryos (MD). The results are presented with 95% confidence intervals (CI) under a random effects model.

Eight studies (*n* = 983) reported on the number of oocytes retrieved. Pooled analysis showed no significant difference between the melatonin and control groups (SMD = 0.45, 95% CI: −0.04 to 0.94; random-effects model). Substantial heterogeneity was observed (*I*^2^ = 86.1%, *p* < 0.0001) (Figure 3A). Sensitivity analysis identified the study by Sadeghpour et al. (19) as a major source of heterogeneity. Excluding this study reduced the effect size to SMD = 0.17 (95% CI: −0.04 to 0.38) and decreased heterogeneity (*I*^2^ = 38.0%, *p* = 0.1051) (Supplementary Figure S2A). Visual inspection of the funnel plot did not suggest substantial publication bias (*t* = 1.57, *p* = 0.1515) (Supplementary Figures S3A, S4A).

### Number of MII oocytes

Six studies (*n* = 418) reported MII oocyte counts. Melatonin supplementation significantly increased the number of MII oocytes (SMD = 0.99, 95% CI: 0.29 to 1.69; random-effects model), though heterogeneity was high (*I*^2^ = 88.2%, *p* < 0.0001) (Figure 3B). When high risk-of-bias studies were excluded, the effect remained significant (SMD = 1.21, 95% CI: 0.20–2.21), but heterogeneity persisted (*I*^2^ = 90.7%) (Supplementary Table S5). Excluding the Sadeghpour study reduced the effect to SMD = 0.63 (95% CI: 0.41 to 0.84) and heterogeneity to *I*^2^ = 45.5% (*p* = 0.1188) (Supplementary Figure S2B). Visual inspection of the funnel plot suggested potential publication bias, particularly before exclusion of the Sadeghpour study (Supplementary Figure S3B).

### Fertilization rate

Four studies (*n* = 275) evaluated fertilization rate. Meta-analysis showed that melatonin significantly improved fertilization outcomes (OR = 1.32, 95% CI: 1.01 to 1.73; random-effects model). Moderate heterogeneity was detected (*I*^2^ = 72.6%, *p* = 0.0013) (Figure 3C). After excluding high risk-of-bias studies, the effect was attenuated and became non-significant (OR = 1.28, 95% CI: 0.91–1.80), with heterogeneity remaining moderate (*I*^2^ = 74.2%) (Supplementary Table S5). Stratified analyses suggested that COS protocols contributed to heterogeneity, with *I*^2^ reduced to 3.9% in specific subgroups. Visual inspection of the funnel plot did not indicate substantial publication bias (Supplementary Figure S3C).

### Number of high-quality embryos

Four studies (*n* = 456) reported high-quality embryo counts. Melatonin significantly increased the number of high-quality embryos (MD = 0.43, 95% CI: 0.07 to 0.79; fixed-effects model). Heterogeneity was low (*I*^2^ = 10.3%, *p* = 0.3500) (Figure 3D). Visual inspection of the funnel plot did not suggest substantial publication bias (Supplementary Figure S3D).

### Subgroup analyses of supplement melatonin additionally

Multidimensional subgroup analyses were performed to explore sources of heterogeneity (Supplementary Tables S2–S4). Stratification by COS protocol showed that melatonin significantly improved clinical pregnancy rates in patients receiving long GnRH agonist protocols (OR = 1.41, 95% CI: 1.04–1.90) and in IUI populations (OR = 2.17, 95% CI: 1.06–4.11). However, oocyte-related outcomes remained highly heterogeneous (*I*^2^ > 90%) within the long GnRH-a subgroup. Dose-based analysis revealed that ≤3 mg melatonin significantly improved clinical pregnancy (OR = 1.59, 95% CI: 1.22–2.07) and high-quality embryo formation (MD = 0.48, 95% CI: 0.09–0.87), with low heterogeneity. By contrast, studies using doses >3 mg showed considerable variability (*I*^2^ > 80%) and yielded inconsistent results, though the limited number of such trials and differences in study design may partly explain these findings. Additionally, analysis stratified by patient population showed that melatonin had the most consistent benefits in women with PCOS and diminished ovarian reserve (DOR), particularly for clinical pregnancy, oocyte quality, and embryo outcomes.

## Discussion

This systematic review included 11 randomized controlled trials (RCTs) encompassing a total of 1,481 infertile women and evaluated the efficacy of melatonin supplementation in assisted reproductive technology (ART). Our meta-analysis demonstrated that melatonin significantly improves clinical pregnancy rates (OR = 1.59, 95% CI: 1.22–2.07) and the number of high-quality embryos (MD = 0.43, 95% CI: 0.07–0.79). These findings support its potential clinical utility in enhancing ART outcomes. While an upward trend was observed in the number of retrieved oocytes, the difference did not reach statistical significance. This discrepancy may be attributed to methodological heterogeneity, including variations in trial design, patient demographics, and melatonin dosing regimens.

Melatonin, a neuroendocrine hormone, is well known for its roles in circadian rhythm regulation, antioxidant defense, and anti-aging mechanisms (25, 26). These physiological functions provide a compelling rationale for its use in ART. Notably, prior studies have highlighted the associations between sleep quality, oxidative stress, and reproductive outcomes. Improved sleep has been linked to increased clinical pregnancy rates (27), while antioxidant supplementation has been shown to enhance oocyte yield and embryo quality, and reduce gonadotropin requirements—ultimately improving ART efficacy (28).

Building upon this mechanistic foundation, our meta-analysis confirms and extends previous evidence supporting melatonin's reproductive benefits in ART contexts (6, 17). Specifically, we observed a pooled odds ratio for clinical pregnancy (OR = 1.59) that exceeds that reported by Hu et al. (OR = 1.43) (17), likely reflecting the inclusion of more recent trials and stringent methodological criteria. In contrast to prior reviews that reported significant increases in oocyte yield (e.g., MD = 0.98, 95% CI: 0.52–1.44) (17, 28), our analysis did not find a statistically significant effect of melatonin on this outcome. This divergence may be due to the inclusion of multi-ingredient antioxidant supplements in earlier studies (e.g., combinations with myo-inositol, folic acid, or selenium), which confounded the specific contribution of melatonin (29). To address this, we included only trials evaluating melatonin as a standalone intervention, thereby improving the precision of our effect estimates. Subgroup analyses suggested that lower doses (≤3 mg/day) of melatonin consistently improved clinical pregnancy rates and embryo quality. However, the apparent inferiority of higher doses should be interpreted cautiously, as it may reflect differences in study design or patient populations rather than a true biological threshold. Evidence from other fields reinforces this caution: while animal models show broad protective effects (30, 31), clinical data in humans are less consistent. At higher doses, melatonin remains generally safe but has been associated with increased adverse events such as drowsiness, headache, and dizziness (32), and in some contexts even retinal damage (33). These observations suggest that variability at higher doses may relate not only to study design, but also to non-linear pharmacodynamics and tolerability issues in humans.

Melatonin exerts multiple physiological actions that may underlie its reproductive benefits. As a potent endogenous antioxidant, it mitigates mitochondrial reactive oxygen species (ROS) accumulation, which is critical for maintaining oocyte and embryo quality (34). Melatonin activates the Nrf2 signaling pathway, upregulating antioxidant enzymes such as superoxide dismutase (SOD) and catalase (CAT), thereby reducing oxidative damage and apoptosis, particularly in porcine and murine aging oocytes (35, 36). Endocrinologically, melatonin may enhance luteal phase support by modulating progesterone production and delaying luteolysis, thereby improving sheep embryo implantation and survival (37). Indeed, recent evidence indicates that pharmacological interventions and luteal support strategies during transfer cycles can significantly affect outcomes, further underscoring the potential endocrine relevance of melatonin (38, 39).

Beyond clinical data, several mechanisms have been demonstrated only in preclinical models. In animal models, it has also been shown to influence early embryonic development via regulation of IGF-1 and pineal–gonadal axis signaling (40). From a cellular perspective, melatonin promotes cytoplasmic maturation by optimizing the spatial arrangement of intracellular organelles such as the Golgi apparatus and endoplasmic reticulum, ultimately facilitating fertilization and blastocyst development (41). At the molecular level, melatonin modulates key signaling cascades such as the PI3K–AKT–FOXO3 pathway, delaying follicular activation and ovarian aging (42). In PCOS models, it has also been shown to improve uterine redox balance and energy metabolism, further supporting fertility (43).

This review offers several methodological strengths. We conducted a comprehensive literature search, included only RCTs, and applied rigorous criteria for data extraction and synthesis. Outcomes assessed encompassed both clinical endpoints (e.g., pregnancy rates) and laboratory markers (e.g., MII oocyte count, embryo quality). Random-effects models were employed to account for between-study heterogeneity, and sensitivity analyses confirmed the robustness of our findings. Publication bias appeared minimal, based on visual inspection of funnel plots.

Nonetheless, limitations should be acknowledged. Several included trials had small sample sizes, and outcomes such as fertilization rate and high-quality embryo count were reported in a limited subset of studies, increasing the risk of imprecision. Moreover, residual confounding from factors such as age, ovarian reserve, endocrine status, and lifestyle cannot be ruled out. Moreover, residual confounding from factors such as age, ovarian reserve, endocrine status, and lifestyle cannot be ruled out (44, 45). Beyond these factors, baseline uterine characteristics—such as endometrial receptivity and structural factors—can also materially influence implantation and ART success, as highlighted in recent studies (46, 47). In addition, evidence on the long-term or high-dose use of melatonin in reproductive-age women is limited, and potential cumulative effects remain uncertain. Importantly, live birth and ongoing pregnancy outcomes, which are more patient-relevant than clinical pregnancy, were rarely reported across the included trials, thereby limiting the clinical interpretability of our findings. Publication bias assessment was also limited, as several endpoints included fewer than 10 studies and thus relied mainly on visual inspection of funnel plots. Additionally, sensitivity analyses revealed that the pooled results for some endpoints were influenced by individual studies, such as the trial by Sadeghpour et al. (19) and by high risk-of-bias trials, particularly for MII oocytes and fertilization, underscoring the need for further large-scale, high-quality RCTs.

## Conclusion

This meta-analysis consolidates evidence on melatonin's potential to improve intermediate reproductive outcomes, including fertilization, embryo quality, and clinical pregnancy rates, among women undergoing ART. However, evidence regarding definitive outcomes such as live birth and miscarriage remains insufficient. With a favorable safety profile, melatonin could be a low-cost adjunct, particularly for patients with diminished ovarian reserve or oxidative stress-related infertility, though standardized guidelines are lacking. Large-scale RCTs are needed to define optimal regimens, identify responsive subgroups, and clarify its impact on long-term outcomes.

## Data Availability

The original contributions presented in the study are included in the article/Supplementary Material, further inquiries can be directed to the corresponding author/s.
