# Supplementary material for: Melatonin improved the outcomes of women with ART: a systematic review and meta-analysis of randomized trials
Source: Front Reprod Health. 2025 Sep 23;7:1680984. doi: 10.3389/frph.2025.1680984 (PMC12500685; doi:10.3389/frph.2025.1680984)
Supplement: Supplementary file 1 [file Datasheet1.docx]

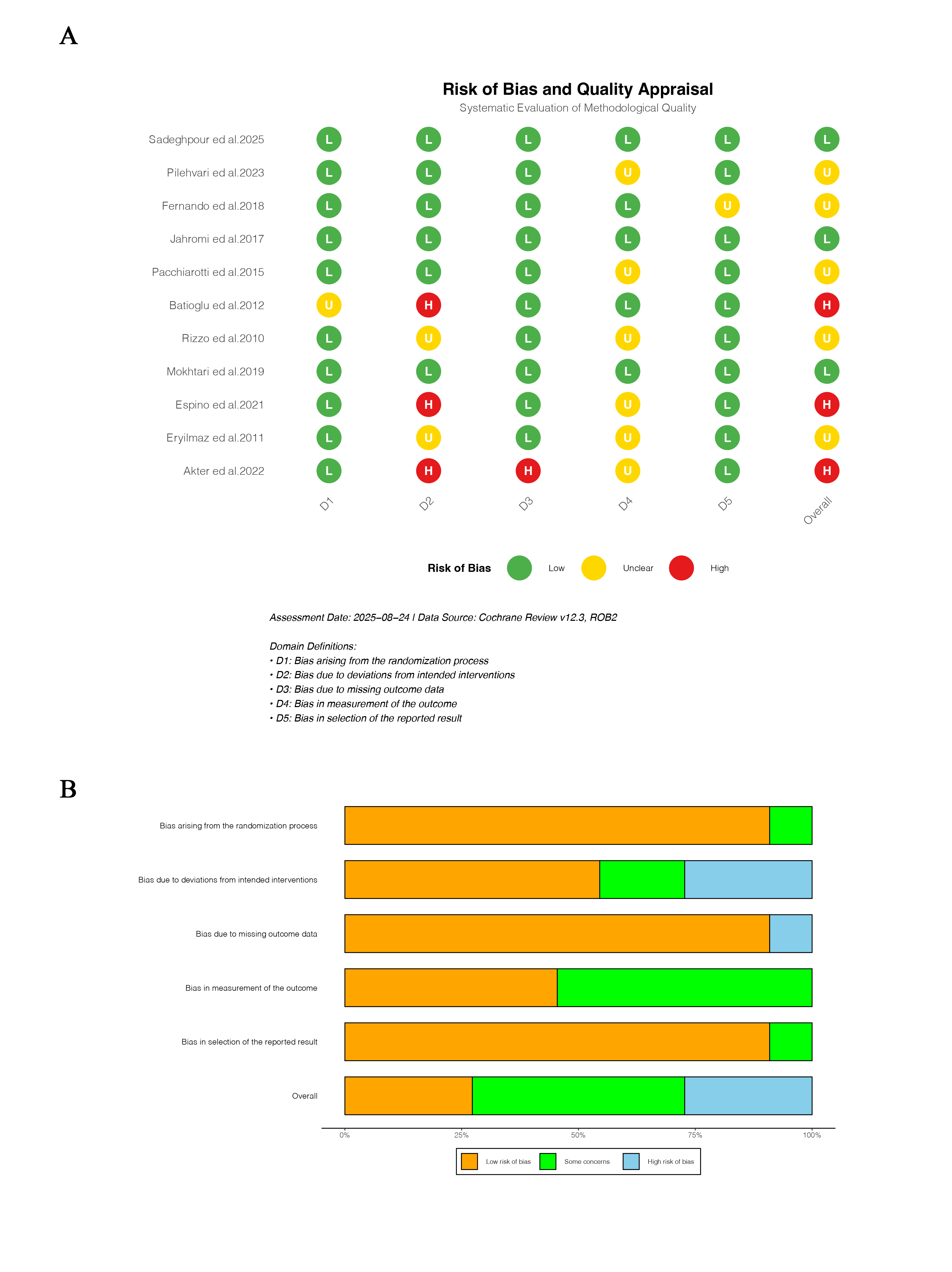


**Supplementary Figure 1.** Risk of bias assessment for the included randomized controlled trials using the Cochrane Risk of Bias tool.

(**A**) Traffic light plot showing the risk of bias judgments for each domain in each study.

(**B**) Summary bar plot showing the proportion of studies rated as low, unclear, or high risk of bias for each domain.


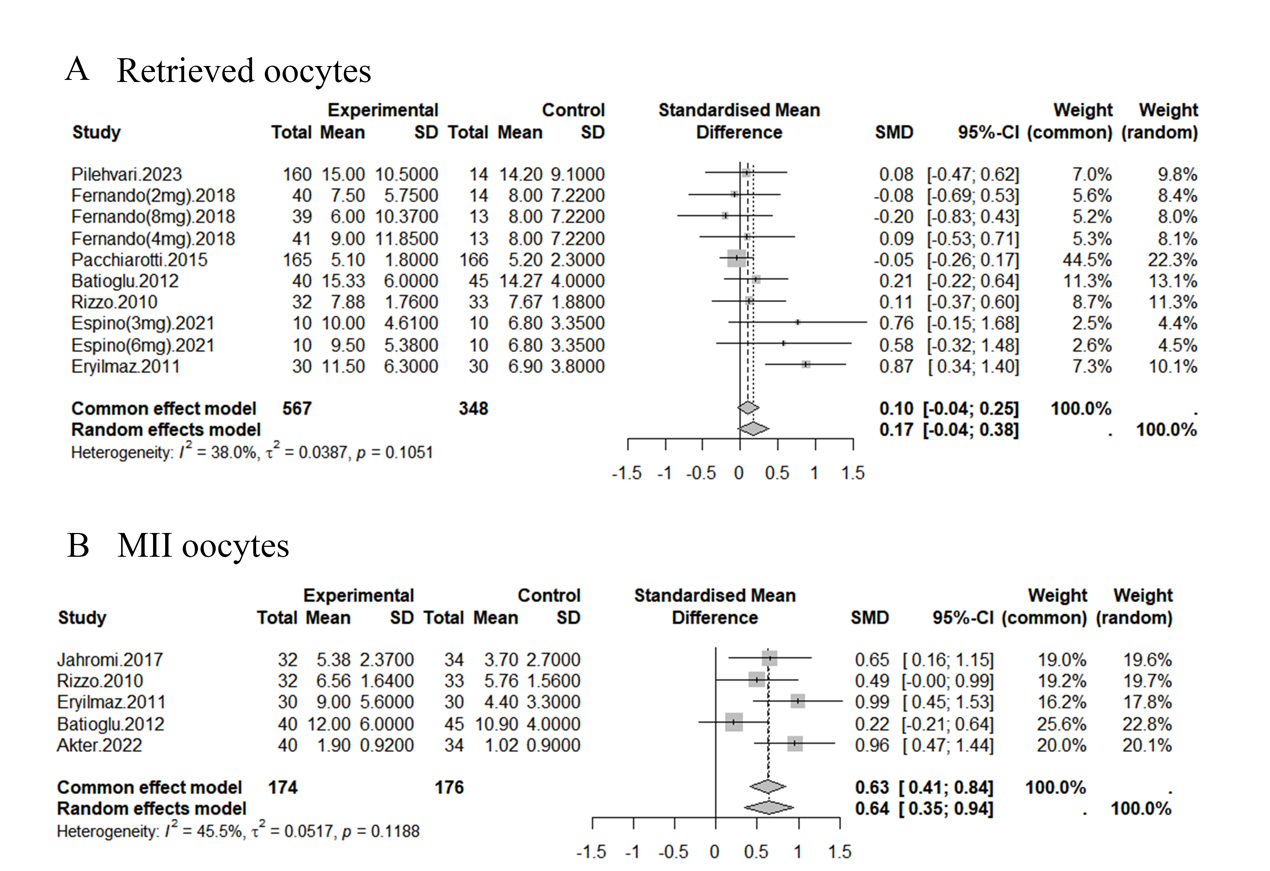


**Supplementary Figure 2.** Funnel plots evaluating potential publication bias in studies assessing the effect of melatonin supplementation on oocyte outcomes. (**A**) Number of retrieved oocytes. (**B**) Number of MII (metaphase II) oocytes. The vertical line indicates the overall standardized mean difference (SMD), and the diagonal lines represent pseudo 95% confidence limits. Studies with high heterogeneity were excluded (Sadeghpour et al.).


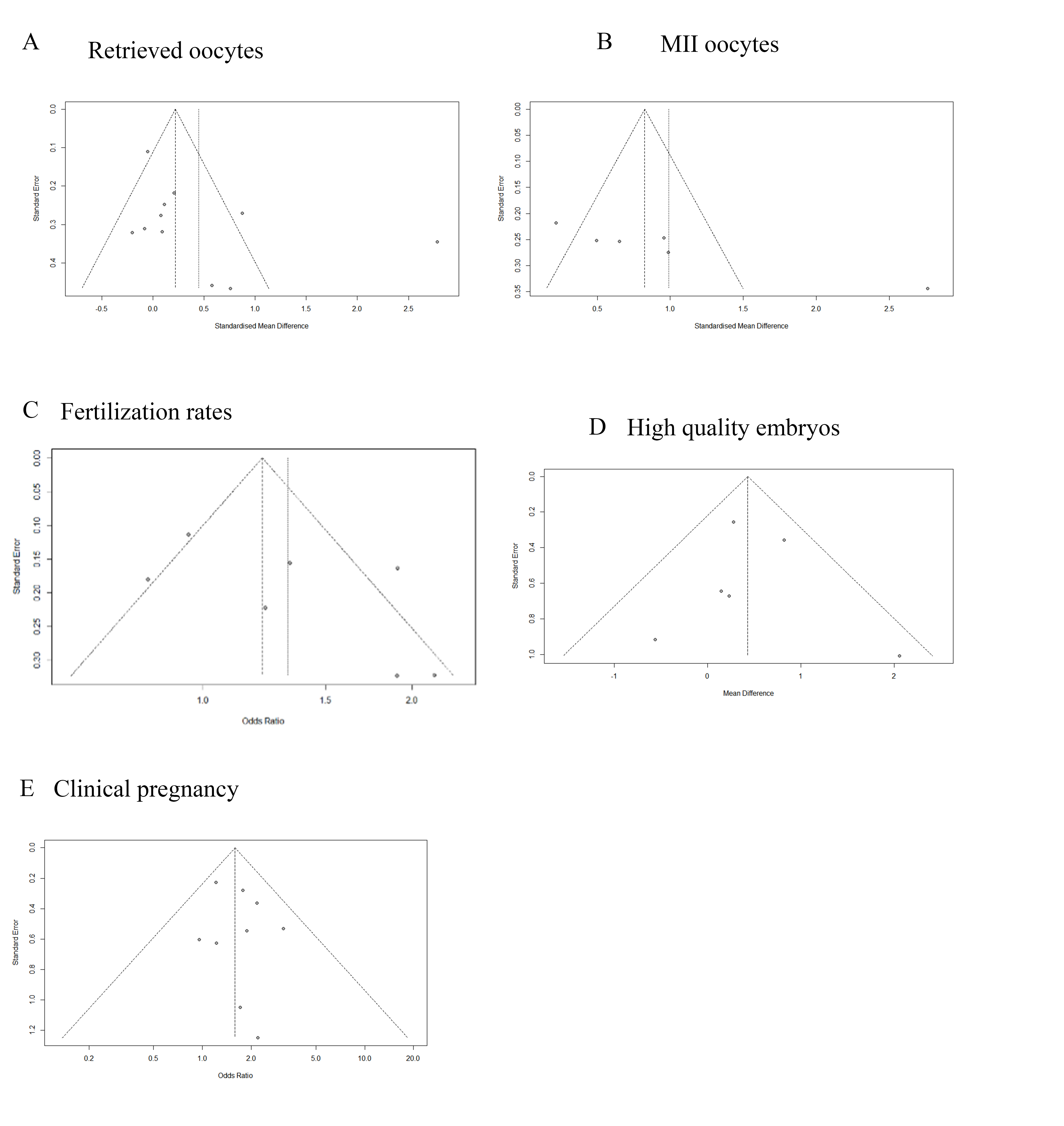


**Supplementary Figure 3.** Funnel plots assessing publication bias in studies investigating the effect of melatonin supplementation on oocyte, embryo, and pregnancy outcomes in assisted reproduction. (**A**) Number of retrieved oocytes. (**B**) Number of MII (metaphase II) oocytes. (**C**) Number of fertilized oocytes. (**D**) Number of high-quality embryos. (**E**) Clinical pregnancy rate. Each plot displays the standard error against the effect size (standardized mean difference [SMD] or odds ratio [OR]) for each included study. The vertical line represents the pooled effect estimate, and the dashed lines indicate the 95% pseudo–confidence intervals.


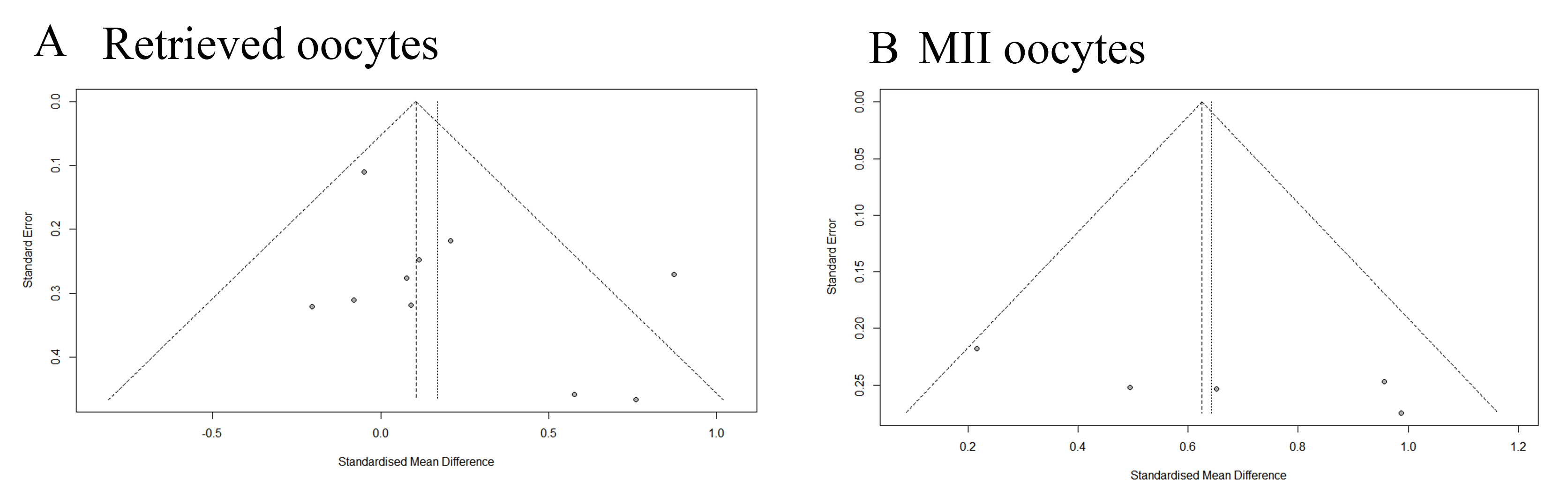


**Supplementary Figure 4.** Funnel plots evaluating potential publication bias in studies assessing the effect of melatonin supplementation on oocyte outcomes. (**A**) Number of retrieved oocytes. **(B**) Number of MII (metaphase II) oocytes. The vertical line indicates the overall standardized mean difference (SMD), and the diagonal lines represent pseudo 95% confidence limits. Studies with high heterogeneity were excluded (Sadeghpour et al.).
