# Supplementary material for: Melatonin improved the outcomes of women with ART: a systematic review and meta-analysis of randomized trials
Source: Front Reprod Health. 2025 Sep 23;7:1680984. doi: 10.3389/frph.2025.1680984 (PMC12500685; doi:10.3389/frph.2025.1680984)
Supplement: Supplementary file 2 [file Datasheet2.docx]

**Supplementary Table 1.** The Pubmed search strategies.

| **Melatonin:** ("melatonin"[MeSH Terms]) OR (Melatonin[Title/Abstract]) OR (ramelteon[Title/Abstract]) OR (rozerem[Title/Abstract]) OR (circadin[Title/Abstract]) OR (agomelatine[Title/Abstract]) OR (valdoxan[Title/Abstract]) OR (melitor[Title/Abstract]) OR (thymanax[Title/Abstract]) OR (tasimelteon[Title/Abstract]) OR (hetlioz[Title/Abstract]) OR (melatonergic[Title/Abstract]) OR (melatonin agonist[Title/Abstract]) OR (5 methoxy n acetyltryptamine[Title/Abstract]) OR (melatonina[Title/Abstract]) OR (melovine[Title/Abstract]) OR (n acetyl 5 methoxytryptamine[Title/Abstract]) OR (slenyto[Title/Abstract]) |
| --- |
| **In vitro fertilization:** "fertilization in vitro"[MeSH Terms] OR "in vitro fertilization"[Title/Abstract] OR "in vitro fertilizations"[Title/Abstract] OR "test tube fertilization"[Title/Abstract] OR "fertilization test tube"[Title/Abstract] OR (("fertilisability"[All Fields] OR "fertilisable"[All Fields] OR "fertilisation"[All Fields] OR "Fertilization"[MeSH Terms] OR "Fertilization"[All Fields] OR "fertilisations"[All Fields] OR "fertilise"[All Fields] OR "fertilised"[All Fields] OR "fertilisers"[All Fields] OR "fertilizers"[Pharmacological Action] OR "fertilizers"[MeSH Terms] OR "fertilizers"[All Fields] OR "fertilises"[All Fields] OR "fertilising"[All Fields] OR "Fertilizations"[All Fields] OR "fertilize"[All Fields] OR "fertilized"[All Fields] OR "fertiliser"[All Fields] OR "fertilizer"[All Fields] OR "fertilizes"[All Fields] OR "fertilizing"[All Fields]) AND "Test-Tube"[Title/Abstract]) OR "test tube fertilization"[Title/Abstract] OR ("Test-Tube"[All Fields] AND "Fertilizations"[Title/Abstract]) OR "fertilizations in vitro"[Title/Abstract] OR "test tube babies"[Title/Abstract] OR (("baby s"[All Fields] OR "babys"[All Fields] OR "infant"[MeSH Terms] OR "infant"[All Fields] OR "Babies"[All Fields]) AND "Test-Tube"[Title/Abstract]) OR (("infant, newborn"[MeSH Terms] OR ("infant"[All Fields] AND "newborn"[All Fields]) OR "newborn infant"[All Fields] OR "Baby"[All Fields] OR "infant"[MeSH Terms] OR "infant"[All Fields]) AND "Test-Tube"[Title/Abstract]) OR "test tube babies"[Title/Abstract] OR "test tube baby"[Title/Abstract] OR ("reproductive techniques, assisted"[MeSH Terms] OR "assisted reproductive technique"[Title/Abstract] OR "reproductive technique assisted"[Title/Abstract] OR (("methods"[MeSH Subheading] OR "methods"[All Fields] OR "Techniques"[All Fields] OR "methods"[MeSH Terms] OR "Technique"[All Fields] OR "technique s"[All Fields]) AND "assisted reproductive"[Title/Abstract]) OR (("methods"[MeSH Subheading] OR "methods"[All Fields] OR "Techniques"[All Fields] OR "methods"[MeSH Terms] OR "Technique"[All Fields] OR "technique s"[All Fields]) AND "assisted reproductive"[Title/Abstract]) OR "assisted reproductive technics"[Title/Abstract] OR "assisted reproductive technic"[Title/Abstract] OR ((("reproduction"[MeSH Terms] OR "reproduction"[All Fields] OR "reproductions"[All Fields] OR "Reproductive"[All Fields] OR "reproductively"[All Fields] OR "reproductives"[All Fields] OR "reproductivity"[All Fields]) AND ("Technic"[All Fields] OR "Technics"[All Fields])) AND "Assisted"[Title/Abstract]) OR ((("reproduction"[MeSH Terms] OR "reproduction"[All Fields] OR "reproductions"[All Fields] OR "Reproductive"[All Fields] OR "reproductively"[All Fields] OR "reproductives"[All Fields] OR "reproductivity"[All Fields]) AND ("Technic"[All Fields] OR "Technics"[All Fields])) AND "Assisted"[Title/Abstract]) OR (("Technic"[All Fields] OR "Technics"[All Fields]) AND "assisted reproductive"[Title/Abstract]) OR (("Technic"[All Fields] OR "Technics"[All Fields]) AND "assisted reproductive"[Title/Abstract]) OR "assisted reproductive techniques"[Title/Abstract] OR (("reproduction"[MeSH Terms] OR "reproduction"[All Fields] OR "reproductions"[All Fields] OR "Reproductive"[All Fields] OR "reproductively"[All Fields] OR "reproductives"[All Fields] OR "reproductivity"[All Fields]) AND "technology assisted"[Title/Abstract]) OR "assisted reproductive technologies"[Title/Abstract] OR "assisted reproductive technology"[Title/Abstract] OR "reproductive technologies assisted"[Title/Abstract] OR "technologies assisted reproductive"[Title/Abstract] OR (("Technology"[MeSH Terms] OR "Technology"[All Fields] OR "Technologies"[All Fields] OR "technology s"[All Fields]) AND "assisted reproductive"[Title/Abstract])) OR ("IVF"[Title/Abstract] OR "ICSI"[Title/Abstract] OR "intra cytoplasmic sperm injection"[Title/Abstract] OR "intracytoplasmic sperm injection"[Title/Abstract] OR "fertilization in vitro"[MeSH Terms]) OR ("subfertility"[Title/Abstract] OR "infertility"[Title/Abstract] OR "reproductive sterility"[Title/Abstract] OR "Sub-Fertility"[Title/Abstract]) OR ("decreased ovarian reserve"[Title/Abstract] OR "diminished ovarian reserve"[Title/Abstract]) OR ("ovarian ageing"[Title/Abstract] OR "ovarian aging"[Title/Abstract] OR "ovarian dysfunction"[Title/Abstract] OR "ovarian senescence"[Title/Abstract]) OR ("primary ovarian insufficiency"[MeSH Terms] OR "primary ovarian insufficiency"[Title/Abstract] OR "ovarian insufficiency primary"[Title/Abstract] OR "ovarian failure premature"[Title/Abstract] OR "premature ovarian failure"[Title/Abstract] OR "gonadotropin resistant ovary syndrome"[Title/Abstract] OR "gonadotropin resistant ovary syndrome"[Title/Abstract] OR "resistant ovary syndrome"[Title/Abstract] OR ((("Hypergonadotropic"[All Fields] OR "hypergonadotropism"[All Fields]) AND ("Ovarian"[All Fields] OR "ovarians"[All Fields]) AND ("Failure"[All Fields] OR "failures"[All Fields])) AND "X-Linked"[Title/Abstract]) OR ((("Hypergonadotropic"[All Fields] OR "hypergonadotropism"[All Fields]) AND ("Ovarian"[All Fields] OR "ovarians"[All Fields]) AND ("Failure"[All Fields] OR "failures"[All Fields])) AND "X-Linked"[Title/Abstract]) OR (("genes, x linked"[MeSH Terms] OR ("genes"[All Fields] AND "X-Linked"[All Fields]) OR "x-linked genes"[All Fields] OR "X-Linked"[All Fields]) AND "hypergonadotropic ovarian failure"[Title/Abstract]) OR (("genes, x linked"[MeSH Terms] OR ("genes"[All Fields] AND "X-Linked"[All Fields]) OR "x-linked genes"[All Fields] OR "X-Linked"[All Fields]) AND "hypergonadotropic ovarian failure"[Title/Abstract]) OR (("menopause, premature"[MeSH Terms] OR ("menopause"[All Fields] AND "Premature"[All Fields]) OR "premature menopause"[All Fields] OR ("Premature"[All Fields] AND "Ovarian"[All Fields] AND "Failure"[All Fields]) OR "premature ovarian failure"[All Fields]) AND "X-Linked"[Title/Abstract]) OR (("menopause, premature"[MeSH Terms] OR ("menopause"[All Fields] AND "Premature"[All Fields]) OR "premature menopause"[All Fields] OR ("Premature"[All Fields] AND "Ovarian"[All Fields] AND "Failure"[All Fields]) OR "premature ovarian failure"[All Fields]) AND "X-Linked"[Title/Abstract]) OR "fragile x associated primary ovarian insufficiency"[Title/Abstract] OR "fragile x associated primary ovarian insufficiency"[Title/Abstract] OR (("Fragile"[All Fields] AND "X"[All Fields]) AND "premature ovarian failure"[Title/Abstract]) OR "fmr1 related primary ovarian insufficiency"[Title/Abstract] OR "fmr1 related primary ovarian insufficiency"[Title/Abstract] OR "primary ovarian insufficiency fragile x associated"[Title/Abstract] OR "primary ovarian insufficiency fragile x associated"[Title/Abstract]) OR (("respon"[Title/Abstract] OR "ovarian reserve"[Title/Abstract]) AND ("poor"[Title/Abstract] OR "low"[Title/Abstract] OR "slow"[Title/Abstract] OR "inadequate"[Title/Abstract] OR "suboptimal"[Title/Abstract] OR "decreas*"[Title/Abstract] OR "diminish*"[Title/Abstract])) |
| **Randomized controlled trial:** (("randomized controlled trial"[Title/Abstract]) OR (controlled[Title/Abstract] AND clinical[Title/Abstract] AND trial [Title/Abstract]) OR (((random[Title/Abstract] AND allocation [Title/Abstract]) OR double-blind[Title/Abstract] OR singled-blind[Title/Abstract] OR placebo[Title/Abstract] OR randomly[Title/Abstract] OR randomized[Title/Abstract] OR clinical [Title/Abstract]) AND trial [Title/Abstract]) OR (rct [Title/Abstract])) |

**Supplementary Table 2.** Subgroup analysis of melatonin supplementation stratified by controlled ovarian stimulation (COS) protocol.

| **Subgroup** | **No. of studies** | **No. of women** | **Effect estimate OR/MD/SMD (95% CI)** | **I²** | **p** |
| --- | --- | --- | --- | --- | --- |
| **Clinical pregnancy rate (%)** |  |  |  |  |  |
| GnRH-a long | 6 | 927 | 1.41(1.04;1.90) | 0% | 0.85 |
| GnRH-a short | 1 | 74 | 3.16(1.12;8.92) | N | N |
| GnRH-A | 1 | 40 | 1.71(0.40;7.34) | N | N |
| IUI | 1 | 194 | 2.17(1.06;4.11) | N | N |
| **Number of retrieved oocytes** |  |  |  |  |  |
| GnRH-a long | 5 | 609 | 0.76(-0.24;1.76) | 94.0% | <0.0001 |
| GnRH-A | 2 | 200 | 0.11(-0.20;0.42) | 7.2% | 0.37 |
| **Fertilized oocyte rate(%)** |  |  |  |  |  |
| GnRH-a long | 2 | 150 | 1.00(0.82;1.32) | 3.9% | 0.31 |
| GnRH-A | 2 | 180 | 1.48(1.04;2.10) | 72.6% | 0.01 |
| **Number of MII oocytes** |  |  |  |  |  |
| GnRH-a long | 5 | 344 | 1.00(0.14,1.86) | 90.5% | <0.0001 |
| GnRH-a short | 1 | 74 | 0.96(0.47;1.44) | N | N |
| **High quality embryo count** |  |  |  |  |  |
| GnRH-a long | 3 | 216 | 0.41(0.01;0.81) | 25.5% | 0.26 |
| GnRH-A | 1 | 240 | 0.52(-0.31;1.34) | N | N |

**Supplementary Table 3.** Subgroup analysis of melatonin supplementation stratified by dosage (≤3 mg vs. >3 mg).

| **Subgroup** | **No. of studies** | **No. of women** | **Effect estimate OR/MD/SMD (95% CI)** | **I²** | **p** |
| --- | --- | --- | --- | --- | --- |
| **Clinical pregnancy rate (%)** |  |  |  |  |  |
| ≤3mg | 9 | 1215 | 1.59(1.22,2.07) | 0% | 0.80 |
| >3mg | 1 | 20 | 1.71(0.22-13.41) | N | N |
| **Number of retrieved oocytes** |  |  |  |  |  |
| ≤3mg | 6 | 729 | 0.04(-0.12;0.20) | 0% | 0.57 |
| >3mg | 3 | 194 | 0.81(-0.53;2.15) | 93.7% | <0.0001 |
| **Fertilized oocyte rate(%)** |  |  |  |  |  |
| ≤3mg | 4 | 211 | 1.14(0.97;1.34) | 59.9% | 0.06 |
| >3mg | 2 | 89 | 1.42(0.81;2.91) | 84.2% | 0.01 |
| **High quality embryo count** |  |  |  |  |  |
| ≤3mg | 4 | 297 | 0.48(0.09;0.87) | 42.7% | 0.16 |
| >3mg | 1 | 159 | 0.19(-0.72;1.10) | N | N |

**Supplementary Table 4.** Subgroup analysis of melatonin supplementation stratified by patient population.

| **Subgroup** | **No. of studies** | **No. of women** | **Effect estimate OR/MD/SMD (95% CI)** | **I²** | **p** |
| --- | --- | --- | --- | --- | --- |
| **Clinical pregnancy rate (%)** |  |  |  |  |  |
| DOR | 1 | 36 | 2.20(0.19;25.52) | N | N |
| PCOS | 4 | 919 | 1.63(1.21;2.19) | 24.4% | 0.26 |
| Infertility | 3 | 170 | 1.46(0.73;2.91) | 0% | 0.86 |
| **Number of retrieved oocytes** |  |  |  |  |  |
| DOR | 1 | 68 | 2.78(2.10;3.46) | N | N |
| PCOS | 2 | 505 | -0.03(-0.23;0.17) | 0% | 0.68 |
| Infertility | 4 | 350 | 0.14(-0.09;0.36) | 0% | 0.61 |
| **Number of MII oocytes** |  |  |  |  |  |
| DOR | 2 | 134 | 1.69(-0.37;3.76) | 95.9% | <0.0001 |
| PCOS | 1 | 74 | 0.96(0.47;1.44) | N | N |
| Infertility | 3 | 110 | 0.54(0.11;0.98) | 58.8% | 0.09 |
| **High quality embryo count** |  |  |  |  |  |
| DOR | 1 | 66 | 0.82(0.12;1.52) | N | N |
| Infertility | 3 | 390 | 0.29(-0.12;0.71) | 0% | 0.41 |

**Supplementary Table 5.** Pooled estimates after excluding studies with high risk of bias.

| Outcome | Values | 95% CI | *I²* |
| --- | --- | --- | --- |
| Retrieved oocytes | SMD = 0.44 | -0.23; 1.11 | 90.0% |
| MI oocytes | SMD = 1.21 | 0.20; 2.21 | 90.7% |
| Fertilization rates | OR = 1.28 | 0.91; 1.80 | 74.2% |
| High quality embryos | SMD = 0.48 | 0.10; 0.86 | 8.2% |
| Clinical pregnancy rate | OR = 1.54 | 1.16; 2.06 | 0.0% |

Note: Pooled effect sizes were recalculated after excluding studies with a moderate to high risk of bias, as suggested by reviewers. SMD = standardized mean difference; OR = odds ratio; CI = confidence interval; I² = inconsistency index indicating heterogeneity across studies.
