# Supplementary figures and images for: Melatonin improved the outcomes of women with ART: a systematic review and meta-analysis of randomized trials
Source: Front Reprod Health. 2025 Sep 23;7:1680984. doi: 10.3389/frph.2025.1680984 (PMC12500685; doi:10.3389/frph.2025.1680984)

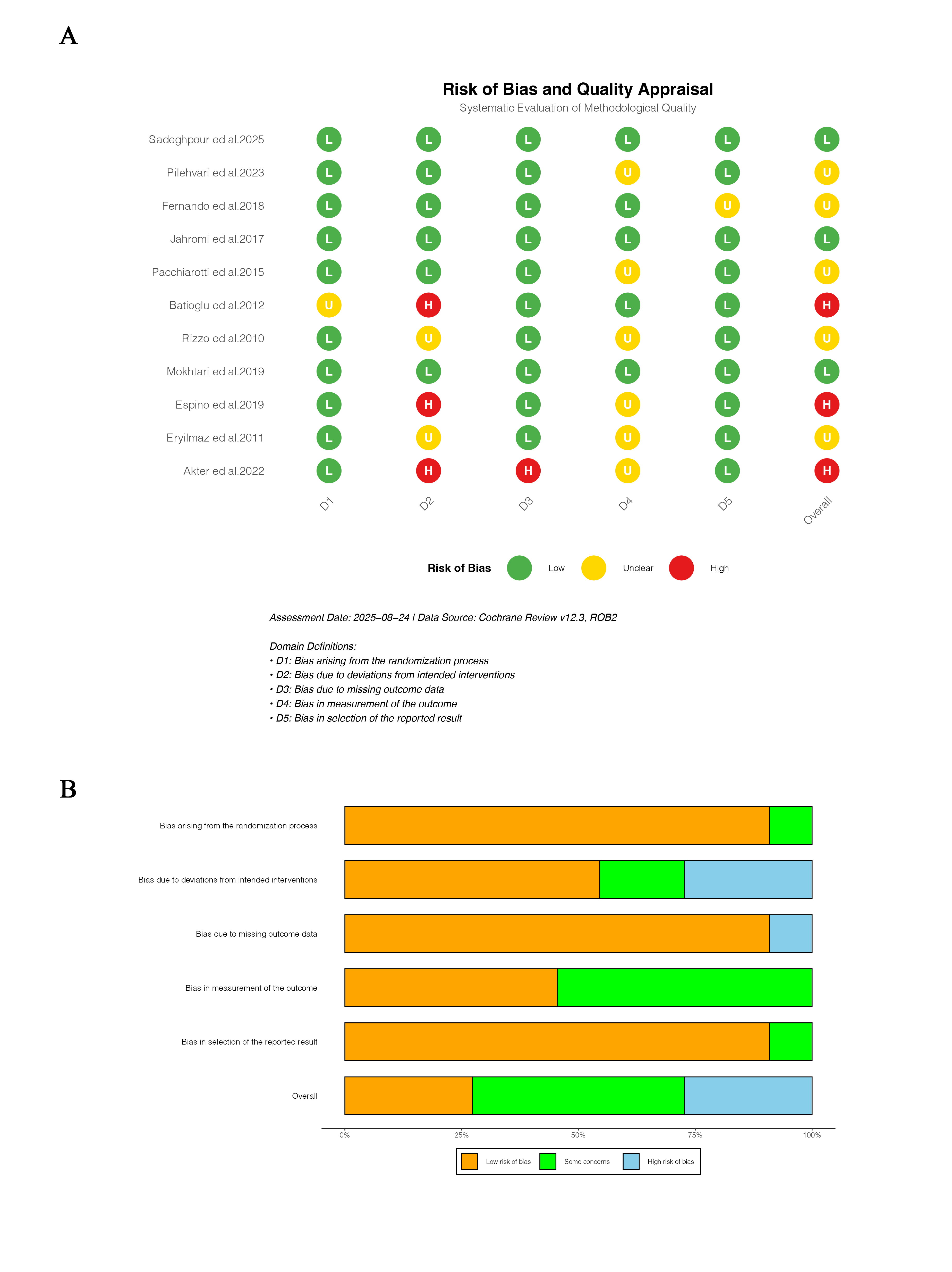

Supplement: Supplementary file 3 [file Image1.tif]

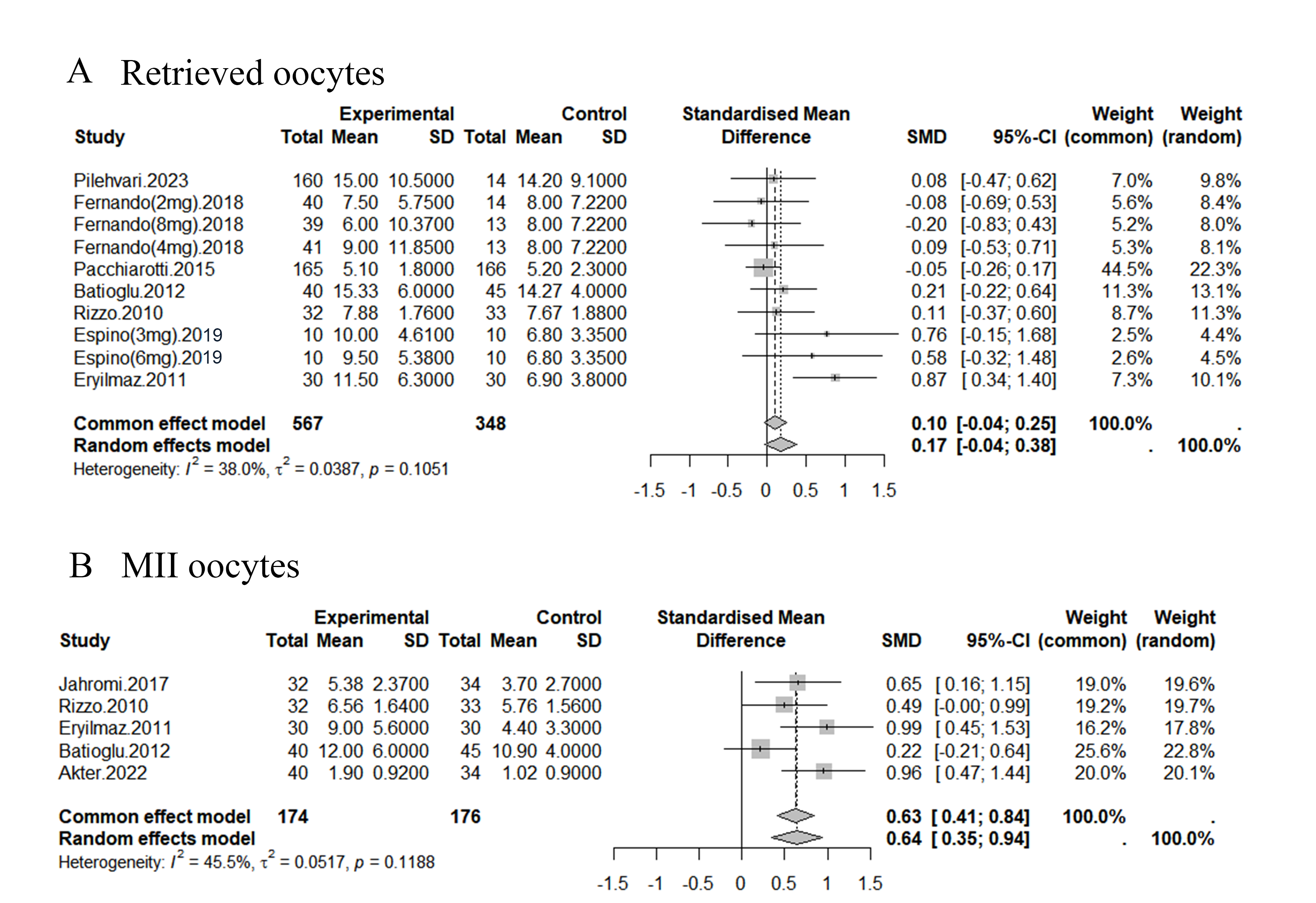

Supplement: Supplementary file 4 [file Image2.tif]
